# Clinical characteristics and image features of pulmonary cryptococcosis: a retrospective analysis of 50 cases in a Chinese hospital

**DOI:** 10.1186/s12890-022-01930-2

**Published:** 2022-04-08

**Authors:** Yuan Lu, Ming Ding, Jing Huang, Cuiping Fu, Yi Wan, Jun Jiang, Jie Huang

**Affiliations:** 1 Department of Respiratory and Critical Care Medicine, Zhongda Hospital, Southeast University, Nanjing, 210009 Jiangsu China; 2 Department of Health Service, Air Force Medical University (Fourth Military Medical University), Xi’an, 710032 Shaanxi China

**Keywords:** Cryptococcosis, Immune function, Clinical features, Imaging characteristics, Cryptococcal capsular antigen test

## Abstract

**Objective:**

To investigate the clinical manifestations and imaging characteristics of pulmonary cryptococcosis, and discuss its guidance in diagnosing.

**Methods:**

The clinical data of patients diagnosed with cryptococcosis in our hospital from January 2014 to May 2020 were collected and retrospectively analyzed. Patients were divided into the immunocompromised group and the immunocompetent group. The symptomatic features, laboratory examination, imaging manifestations, and curative effect were analyzed.

**Results:**

The most common symptoms of patients were cough and sputum production, followed by fever. The immunocompetent group has a significantly higher accident rate of cough and fever than the immunocompromised group, while the immunocompromised group has a significantly higher accident rate of headache and dizziness (*P* < 0.05). The positive rate of serum cryptococcal capsular antigen (CrAg) test of the two groups were 83.33% and 86.96%, respectively. While the positive rate of CrAg test in cerebrospinal fluid of the immunocompromised group was significantly higher than that of the immunocompetent group (*P* < 0.05). The lesions of pulmonary cryptococcosis were predominantly present in the lower part of the lung periphery and significantly distributed in the right lung (*P* < 0.05). The most common imaging finding of pulmonary cryptococcosis was halo sign (64.58%), followed by multiple nodules, and trachea sign was significantly more common in the immunocompetent group.

**Conclusions:**

Cryptococcosis has an insidious onset, which can infect healthy people as well. Conducting a CrAg test is good for screening and diagnosing cryptococcosis. We should be alert for the high risk of cryptococcal meningoencephalitis in patients with compromised immune function.

**Supplementary Information:**

The online version contains supplementary material available at 10.1186/s12890-022-01930-2.

## Background

*Cryptococcus* is a kind of conditional pathogenic fungus that is ubiquitous all over the world. It can cause pulmonary cryptococcosis after inhalation through the respiratory tract, and the infection can easily spread to the central nervous system [1]. Cryptococcosis was previously thought to occur in people with an abnormal immune function such as acquired immune deficiency syndrome (AIDS), but recent studies have shown that as many as 60% of patients with cryptococcosis have normal immune function, so ordinary people are also susceptible to cryptococcosis [2]. The clinical manifestations of cryptococcosis lack specificity and the imaging manifestations are sometimes difficult to distinguish from lobar pneumonia, tuberculosis, tumors or other fungal infections. Only a certain understanding of the disease can be diagnosed promptly, otherwise, it can be prone to missed diagnosis and misdiagnosis [3, 4]. In this study, we summarized the clinical data of 50 patients diagnosed with cryptococcosis in the Zhongda Hospital of Southeast University from January 2014 to May 2020, and analyzed them, hoping to provide strong support for improving clinical diagnosis capabilities.

## Materials and methods

### Research objects

Fifty patients diagnosed with cryptococcosis in the Zhongda Hospital of Southeast University from January 2014 to May 2020 were included in the study, including 28 males and 22 females. Inclusion criteria [5, 6]: Diagnosis refers to the requirements of “Experts Consensus on Diagnosis and Treatment of Cryptococcal Disease in Zhejiang Province” and “Expert Consensus on Diagnosis and Treatment of Cryptococcal Meningoencephalitis”, with clear pathological results or typical imaging findings, positive serum cryptococcal antigen test, tissue specimens stained positively, and patients with intracranial infection have cerebrospinal fluid (CSF) etiology or brain tissue pathology evidence. Include patients with malignant tumors, AIDS, diabetes or autoimmune diseases, taking immunosuppressants, glucocorticoids, etc. into the immunocompromised group, and the remaining patients into the immunocompetent group. Exclusion criteria: patients younger than 16 years old, pregnant women, people suffering from mental or nervous system diseases and unable to take care of themselves. This study was approved by the ethics committee of our hospital (2020ZDSYLL101-P01).

## Research methods

A retrospective analysis of the clinical data of 50 patients, including gender, age, comorbidities, whether to use immunosuppressor or glucocorticoids, first symptoms; laboratory indicators: blood routine examination, C-reactive protein (CRP), serum procalcitonin (PCT), erythrocyte sedimentation rate (ESR); imaging characteristics; treatment drugs, treatment duration and efficacy, etc. The evaluation criteria for curative effect are based on the literature [5, 6], with certain modifications: Effective: the patient survives during the observation period, the symptoms and signs related to the disease are all relieved, the CSF routine and biochemical are normal, the CrAg turns negative, and imaging shows that the lesion completely absorbed or fibrosis after absorption, stable for a long time without progress. Ineffective: disease-related symptoms and signs aggravate or worsen, cultures from CSF or other infection sites continue to be positive, CrAgs continue to be positive for *cryptococcus*, imaging shows that the original lesions are enlarged or new lesions appear; Death: Deaths from all causes directly or indirectly related to cryptococcal infection.

### Statistical methods

All data statistics used are carried out by SPSS22.0 software. Normally distributed measurement data is represented by ${\overline{\text{X}}}\, \pm \,{\text{S}}$, using two independent samples *t* test; non-normally distributed data using median (M) and interquartile range (Q3–Q1) indicates that the Mann–Whitney *U* test is used; the count data is expressed by n (%), and the *χ*^*2*^ test or Fisher’s exact probability test is used. *P* < 0.05 indicates that the difference is statistically significant.

## Results

### General information

Among 50 patients, 43 cases (86%) were diagnosed with cryptococcal pneumonia, of which 5 cases (11.6%) had cryptococcal meningoencephalitis, and another 2 cases (4%) of patients with cryptococcal meningoencephalitis showed no abnormalities in chest imaging. There were 24 patients in the immunocompromised group, including 14 males and 10 females, aged from 27 to 82 years old, with an average age of 58.08 ± 12.29 years old. In terms of underlying diseases, 13 patients had diabetes (1 case with liver cirrhosis and fatty disease). Five cases of autoimmune diseases (1 case of Behcet's disease, 2 cases of lupus erythematosus, 1 case of rheumatoid arthritis with Sjogren’s syndrome, and 1 case of dermatomyositis) were all given oral immunosuppressants or glucocorticoids, and 6 cases of malignant tumors (colon cancer, breast cancer, esophageal cancer, pancreatic cancer, mucinous adenocarcinoma, angioimmunoblastic T-cell lymphoma combined with appendix intraepithelial carcinoma (1 case each), 2 cases of nephropathy (1 case each for IgA nephropathy and 1 case for nephrotic syndrome) and 1 case of AIDS. There were 26 patients in the immunocompetent group, including 14 males and 12 females, aged 21–84 years, with an average age of 45.23 ± 13.82 years. There was no significant difference between the two groups of patients in gender and age (*P* > 0.05). The most common complaints of patients were cough and sputum (22/50, 44%), 13 cases (26%) had a fever, 7 cases (14%) had headache and dizziness, and 6 cases (12%) had chest pain and chest tightness, 2 patients (4%) complained of vomiting and hiccups, and 14 patients (28%) had no symptoms. The incidence of cough, sputum and fever in the immunocompetent group was significantly higher than that of the immunocompromised group, and the incidence of headache and dizziness in the immunocompromised group was significantly higher than that of the immunocompetent group (*P* < 0.05) (Table 1).

**Table 1:** Comparison of clinical characteristics between two groups of patients with cryptococcosis [n(%)]

Item	Group		t/χ^2^	*P*
Normal information	Immunocompromised group (n = 24)	Immunocompetent group (n = 26)		
Gender (male/female)	14/10	14/12	0.102	0.749
Age (years)	58.08 ± 12.29	45.23 ± 13.82	3.463	0.590
First symptoms				
Cough, expectoration	5 (20.83)	17 (65.38)	10.052	0.002
Fever	3 (12.5)	10 (38.46)	10.940	0.002^a^
Headache, dizziness	6 (25)	1 (3.85)	4.638	0.045^a^
Chest pain, chest tightness	2 (8.33)	4 (15.38)	0.588	0.669^a^
Vomiting, hiccups	2 (8.33)	0		
Asymptomatic	9 (37.5)	5 (19.23)	2.066	0.151

^a^Fisher exact probability method

### Laboratory inspection

Laboratory examination results showed that the patient's peripheral white blood cells (WBCs), neutrophil and lymphocyte counts were generally within the normal range. Seven cases (14%) had increased white blood cells and neutrophils, 4 cases (8%) had decreased white blood cells, and 2 cases (4%) had decreased neutrophils,13 cases (26%) showed decreased lymphocytes (8 immunocompromised and 5 immunocompetent). Only 1 case (2%) showed an increase in lymphocytes. There was no significant difference between the two groups of patients in the counts of WBCs, neutrophils, lymphocytes, procalcitonin (PCT), C-reactive protein (CRP) and erythrocyte sedimentation rate (ESR) (*P* > 0.05). Forty-seven patients underwent CrAg detection. Of 24 patients in the immunocompromised group, 20 (83.33%) were positive, and 20 of 23 patients (86.96%) in the immunocompetent group were positive. There was no significant difference between the two groups (*P* > 0.05). A total of 17 patients underwent CSF CrAg test, 7 cases were positive, including 6 out of 8 cases in the immunocompromised group (75%), and 1 out of 9 cases in the immunocompetent group (11.11%), the difference between the two groups was statistically significant (*P* < 0.05) (Table 2).

**Table 2:** Comparison of laboratory findings between two groups of patients with cryptococcosis [n(%)]

Item	Number of cases	Group		t/χ^2^/U	*P*
	(immune deficiency group/immune ability group)	Immunocompromised group	Immunocompetent group		
White blood cell count (× 10^9^/L)	24/26	6.57 ± 3.32	7.16 ± 1.95	3.013	0.089
Number of neutrophils (× 10^9^/L)	24/26	4.69 ± 2.81	4.75 ± 2.062	1.591	0.213
Lymphocyte count (× 10^9^/L)	24/26	1.31 ± 0.81	1.62 ± 0.55	2.547	0.117
PCT (ng/mL)	9/9	0.11 ± 0.07	0.16 ± 0.30	2.950	0.105
CRP (mg/L)	15/21	6.67 (23.28)	6.41 (55.25)	143.000	0.642
ESR (mm/h)	14/15	8.50 (8.50)	9.00 (61.00)	104	0.965
Serum CrAg	24/23	20 (83.33)	20 (86.96)	0.122	0.727^a^
CSF CrAg	8/9	6 (75)	1 (11.11)	7.137	0.015^a^

^a^Fisher exact probability method

### Chest imaging findings

**Fig. 1:**
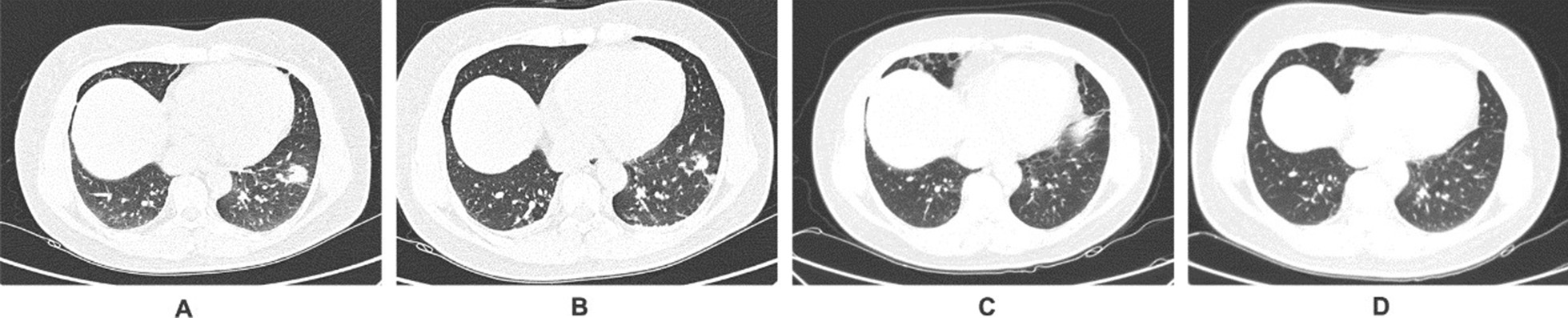
CT image of patient 1 with pulmonary cryptococcosis**.** Patient 1, female, 46 years old, was admitted to the hospital due to chest pain and chest tightness. The treatment period was 6 months. **A** A single nodule on the left lower lobe with fine burrs on the periphery; **B** After 2 months of fluconazole treatment, the lesion has been absorbed, and a cord shadow can be seen; **C** The lesion is almost completely absorbed after 2 and a half months of fluconazole treatment; **D** Chest CT after 32 months treatment, the lesion is completely absorbed

**Fig. 2:**
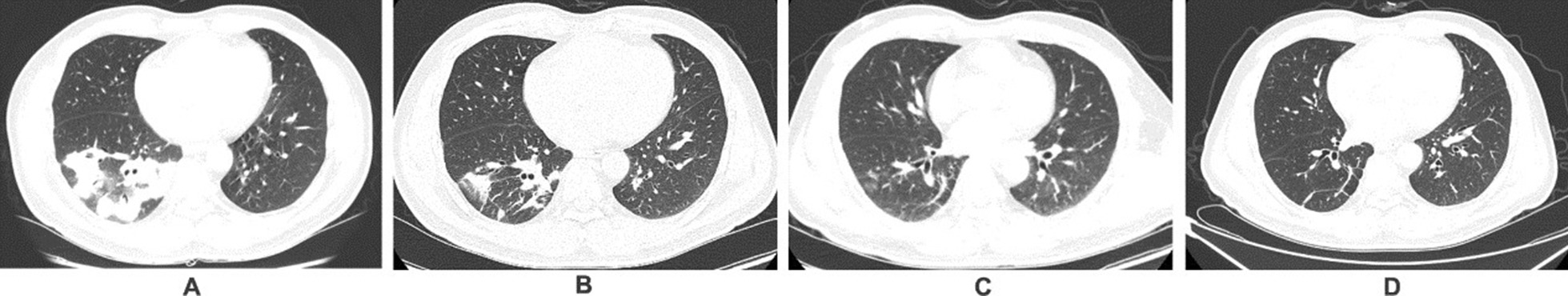
CT image of patient 2 with pulmonary cryptococcosis. Patient 2, male, 53 years old, was admitted to the hospital due to cough. The treatment period was 16 months. **A** There are multiple nodules under the pleura of the right lower lobe, with a tendency to fuse, with signs of halo around it; **B** after fluconazole treatment for 2 months, the lesion has been absorbed, and the cord is visible; **C** after fluconazole treatment for 9 months, the lesion is almost completely absorbed; **D** 39 months later, the lesion in chest CT is completely absorbed, and only a small amount of cord shadow remains

**Fig. 3:**
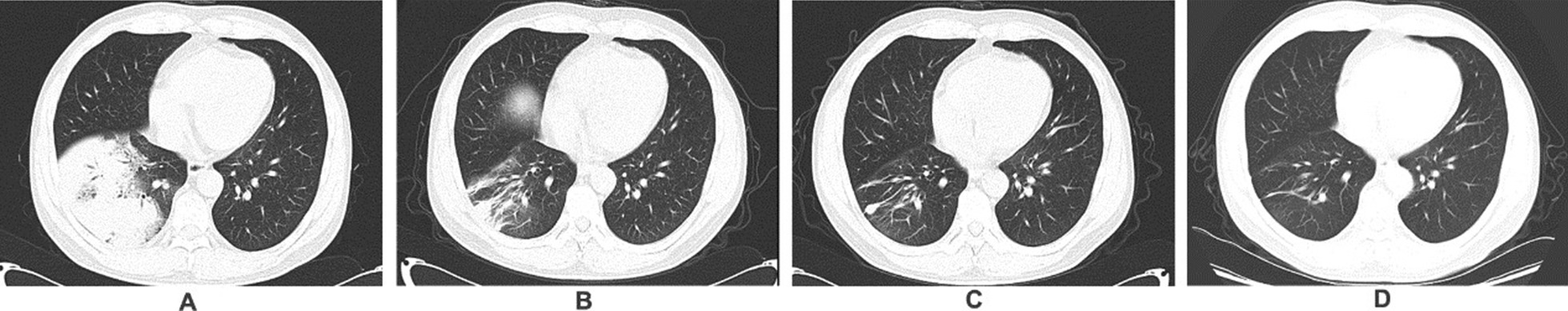
CT image of patient 3 with pulmonary cryptococcosis. Patient 3, male, 42 years old, was admitted to the hospital due to cough. The treatment period was 6 months. **A** Large consolidation of the lower lobe of the right lung, showing signs of bronchial inflation; **B** after fluconazole treatment for 7 weeks, the lesion is absorbed significantly; **C** after fluconazole treatment for 4 months, the lesion is almost completely absorbed, with a striped shadow; **D** chest CT after 28 months, the lesion is completely absorbed, and only a small amount of cord shadow remains

**Fig. 4:**
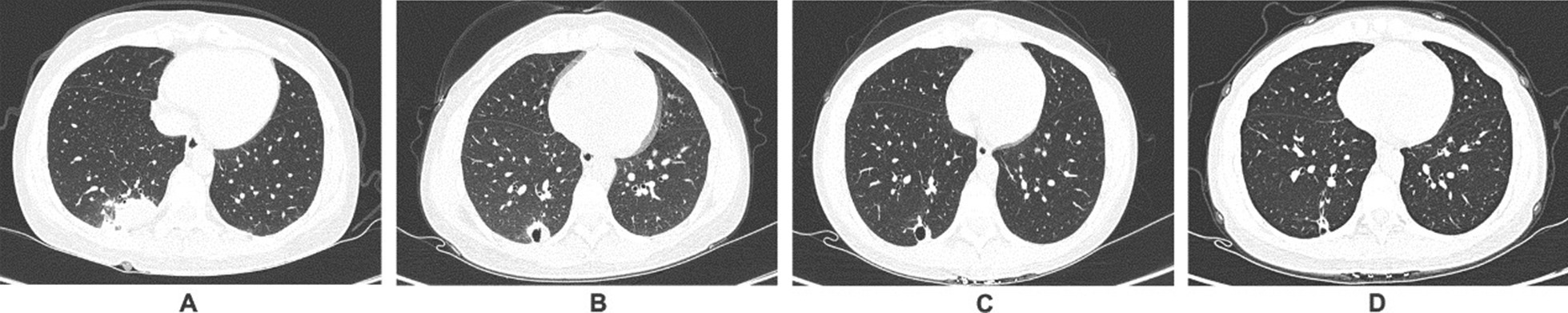
CT image of patient 4 with pulmonary cryptococcosis. Patient 4, female, 29 years old, was admitted to the hospital due to cough. The treatment period was 12 months. **A** Subpleural nodules in the left lower lung, with signs of halo around; **B** after fluconazole treatment for 3 months, the lesion is absorbed, but a cavity appears in the center; **C** after fluconazole treatment for 3 months, the lesion is further absorbed and the cavity wall thinning; **D** after 27 months, chest CT showed that the lesion was completely absorbed, the cavity disappeared, and only a small amount of cord shadow remained

In terms of overall chest imaging, the main features of the patient’s chest imaging are the peri-lobe distribution. Of the 48 patients with lung lesions, 47 (97.92%) have lesions mainly located under the pleura. Seventeen cases have lesions on the left lung, and thirty-eight cases distributed on the right lung, the difference is statistically significant (*χ*^*2*^ = 18.774, *P* = 0.000), but the difference in distribution between the two groups is not statistically significant (*P* > 0.05).Twenty-seven cases (57.45%) are distributed in the left lower lobe, 21 cases (44.68%) are distributed in the right lower lobe, the difference between the two lobes is not statistically significant (*χ*^*2*^ = 1.533, *P* = 0.216). However, the distribution difference between the middle, lower and upper lobes is statistically significant (*χ*^*2*^ = 40.199, *P* = 0.000). In terms of the morphological characteristics of the lesion, the halo sign around the lesion was the most common sign, seen in a total of 31 cases (64.58%). Secondly, a total of 27 cases (56.25%) showed multiple nodular lesions. Thirdly, in 16 cases showing pneumonia-like consolidation, 11 cases (45.83%) belong to the immunocompetent group, which was more than 5 cases (22.72%) in the immunocompromised group, but there was no statistically significant difference between the two (*P* > 0.05). The sign of bronchial inflation in lung lesions was more often present in the immunocompetent group, seen in 13 cases (54.17%), which was significantly more than 2 cases (9.09%) of the immunocompromised group (*P* < 0.05) (Table 3) (Figs. 1, 2, 3, 4).

**Table 3:** Comparison of imaging features of between two groups of patients with cryptococcosis [n(%)]

Feature	Group		χ^2^	*P*
	Immunocompromised group (n = 24)	Immunocompetent group (n = 26)		
*Distributed*				
Central type	0	1 (3.85)		
Peripheral type	22 (91.67)	25 (96.15)	0.446	0.602
No abnormality	2 (8.33)	0		
Left lung	13 (54.17)	14 (53.85)	0.017	0.897
Right lung	18 (75.00)	20 (76.92)	0.025	0.873
Left upper lobe of left lung	3 (12.50)	3 (11.54)	0.011	1.000^a,b^
Lower left leaf	10 (41.67)	11 (42.31)	0.002	0.963^a^
Upper right leaf	4 (16.67)	3 (11.54)	0.273	0.697^a,b^
Lower right leaf	12 (50.00)	15 (57.69)	0.297	0.586^a^
Right middle lobe	2 (8.33)	2 (7.69)	0.007	1.000^a,b^
*Performance*				
Single nodule	6 (25.00)	5 (19.23)	0.262	0.609
Multiple nodules	12 (50.00)	15 (57.69)	0.300	0.584
Pneumonia-like consolidation	4 (16.67)	11 (42.31)	3.994	0.063^b^
Invasive shadow	5 (20.83)	3 (11.54)	0.836	0.451^b^
Cable shadow	4 (16.67)	6 (23.08)	0.314	0.725^b^
Halo sign	12 (50.00)	19 (73.08)	3.166	0.075
Bronchial inflation sign	2 (8.33)	13 (50.00)	10.613	0.001^b^
Pleural effusion	1 (4.17)	2 (7.69)	0.270	1.000^b^
Hollow	1 (4.17)	2 (7.69)	0.270	1.000^b^
Swollen lymph nodes	0	1		

^a^Correction calibration level *P* < 0.00625

^b^Fisher exact probability method

### Method of diagnosis

Of the 50 patients, the diagnosis was obtained by bronchoscopy in 16 cases, percutaneous lung puncture in 13 cases, thoracic surgery in 7 cases, and neurosurgical abscess resection in 1 case. All the above pathological specimens are diagnosed after special stainings such as periodic acid Schiff (PAS), Gomori's methanamine silver (GMS) and mucicarmin. Moreover, 6 cases were diagnosed with special staining of CSF after lumbar puncture, and 7 cases that refused invasive operation were diagnosed by two times of positive blood CrAg test (Additional file 1).

### Treatment plan and efficacy

The two groups of patients were given different treatment plans according to whether they were combined with cryptococcal meningoencephalitis. The patients without cryptococcal meningoencephalitis were mainly treated with fluconazole, and those with cryptococcal meningoencephalitis were treated with amphotericin B and flucytosine. Until the study deadline, the shortest treatment period is 3 months and the longest treatment period is 24 months. The median treatment time in the immunocompromised group was 5.5 (6.75) months and 6 (6) months in the immunocompetent group. The difference was not statistically significant (*U* = 252.5, *P* = 0.154). The median treatment time of patients with or without cryptococcal meningitis was 6 (11) and 6 (6.5) months, retrospectively. There was no statistically significant difference between them (*P* = 0.810). In the immunocompetent group, 1 case diagnosed via bronchoscopy biopsy failed to respond to 3 months of fluconazole treatment, the lesion was finally removed surgically. Another patient in the immunocompromised group who failed to respond to treatment received surgical resection as well, the pathological report showed the mucinous adenocarcinoma. One AIDS patient in the immunocompromised group was lost to follow-up after transfer to another hospital. One patient, whose lesion did not shrink significantly after 3 months of treatment, discontinued treatment by himself. There was one death in each of the two groups, both had cryptococcal meningoencephalitis. The effective rates of the two groups were 83.33% and 92.31%, respectively, and the difference was not statistically significant (*χ*^*2*^ = 0.952, *P* = 0.409) (Table 4).

**Table 4:** The therapeutic methods of two groups of patients with cryptococcosis [n(%)]

Treatment programs	Immunocompromised group (n = 24)		Immunocompetent group (n = 26)		Total
	Incorporate hidden brain (n = 6)	Uncombined hidden brain (n = 18)	Incorporate hidden brain (n = 1)	Uncombined hidden brain (n = 25)	
Fluconazole	0	17	0	24	41
Fluconazole + amphotericin B*	2	0	0	0	2
Flucytosine + amphotericin B	2	0	1	0	3
Three-drug combination^a^	2	0	0	0	2
Itraconazole	1	0	0	0	0
Surgery	1	4	0	2	7
Unused drugs	0	1	0	1	2

Including amphotericin B and amphotericin B liposomes

^a^Fluconazole + amphotericin B + flucytosine

## Discussion

*Cryptococcus* was often considered to be an opportunistic infection, and the lung is the most commonly invaded organ. The susceptible population is mainly immunocompromised patients. The main risk factors for the disease include AIDS, long-term steroid treatment or the use of immunosuppressants, chronic lymphocytic leukemia or organ transplantation, hematological or solid malignant tumors, as well as diabetes, liver cirrhosis, etc. [7, 8]. Recent studies have shown that the incidence of cryptococcosis in healthy people is also increasing year by year, especially in East Asian countries such as Japan and China. In these countries, the vast majority of cases are non-AIDS-related, and more than 50% have no risk factors leading to abnormal immune function [9, 10]. In this study, we only had 1 case (2%) finally diagnosed with AIDS, which may be related to the fact that our hosptial isn’t an infectious disease hospital, and it is also associated with the fact that the prevalence of AIDS in my country is much lower than that in Europe, America and Africa [11]. More than half of the patients have no potential immune suppression or deficiency, which indicates that the possibility of cryptococcal infection in healthy people is also very high.

In addition to being susceptible to respiratory infections, the central nervous system is often easily affected by cryptococcal infections. About 52–61% of patients have cryptococcal meningoencephalitis caused by disseminated infections [12]. The central tropism of *cryptococcus* is related to the expression of specific metalloprotease and urease. These enzymes can facilitate the passage of *cryptococcus* through the blood–brain barrier, thereby leading to immune tolerance in the center, and promoting high-affinity glucose transporter expression to enhance energy supply [13]. Cryptococcal meningoencephalitis is difficult to treat, and a combination of amphotericin B, fluconazole and other medicine is required. Patients with cryptococcal meningoencephalitis always have to experience severe side effects, high possibility of treatment failure, and poor prognosis. Even in Western developed countries, the mortality rate of AIDS-related cryptococcal meningoencephalitis is as high as 15.3%, and non-AIDS patients have an even higher mortality rate due to misdiagnosis or severe comorbidities [9]. In this study, a total of 7 patients with cryptococcal meningoencephalitis were included, including 6 immunocompromised and 1 immunocompetent. The incidence of cryptococcal meningoencephalitis in the immunocompromised group was significantly higher than that of the immunocompetent group. It indicates that the possibility of cryptococcal meningoencephalitis in immunocompromised patients is higher, which needs to be taken seriously. In addition, one patient who died in this study initially presented with fever, headache, and dizziness. His laboratory results showed hyponatremia. After failing to respond to anti-infection and electrolyte supplementation treatments, he was stained positive for *cryptococcus* in CSF by lumbar puncture. Although received amphotericin B combined with fluconazole, he died quickly due to multiple organ dysfunction. This patient does not have risk factors for immunosuppression, which may be the main reason why cryptococcal infection was not considered at the beginning. This case has sounded the alarm for us that facing elderly and frail patients with suspected encephalitis in the humid areas of the south, the CrAg test can be used as a routine examination for admission.

Cryptococcosis lacks specificity in symptoms. In this study, 14 patients (28%) had no clinical symptoms, and most of them were diagnosed during physical examination. In symptomatic cases, cough and sputum are the most common complaints (44%), which is consistent with previous research results[14]. We have also observed that patients generally have less sputum volume, lack of quality characteristics, and no special manifestations such as the “thread-like” sputum exhibited by respiratory *candida* infection. In addition, a total of 13 patients (26%) get fever, but the body temperature of most of them did not exceed 38.5 °C. It is worth noting that previous studies have suggested that most people with normal immune function do not have the disease, and those with normal immune function have fewer clinical symptoms, mild lung disease, and are less likely to develop respiratory failure and systemic spread. It is somewhat different from our study. In this study, the symptoms of fever, cough, and sputum in the immunocompetent group were far more than those in the immunocompromised group. We believe that this may be related to the stronger Th2-type reaction in the patient's body, especially in the inflammation area of respiratory tract [15, 16].

In terms of laboratory tests, the levels of blood routine, CRP, PCT and other inflammatory indexes of the two groups were generally in the normal range, and the difference between them was not statistically significant. Compared with conventional culture and pathological examinations, CrAg detection has the characteristics and advantages of rapidness, simplicity, sensitivity and high specificity. Its positive results have been identified as the diagnostic criteria by the European Cryptococcal Meningoencephalitis Diagnostic Guidelines. It was recommended by the “Consensus of Zhejiang Experts on Pulmonary Cryptococcosis” as a sign of the start of anti-cryptococcal treatment [17]. The vast majority of cases in this study were diagnosed after the test was carried out in our hospital in 2015. To a certain extent, the higher availability of this test in our country might be part of the reason for the increase of the incidence of cryptococcosis in recent years as the cases have been continuously screened out. It should be noted that in this study, 5 cases that confirmed by pathological special staining were negative more than 2 times for CrAg test. And there were another 2 cases that test negative initially convert to positive after re-examination. Therefore, the active search for pathological evidence and repeated testing cannot be ignored in clinical work.

In imaging studies, it is generally considered that the lesions of typical cryptococcal infection are mostly located in the periphery of the lung field and are mainly nodular. Most cases have more than one lesion, and nodules are more inclined to gather in one place, which is the so-called "Mushroom Brothers Sign", and are often accompanied by halo signs around [3]. In this study, most patients had single or multiple nodular lesions, and the incidence of multiple nodules was higher than that of single ones. In addition, the incidence of pneumonia-like consolidation and bronchial inflation in the immunocompetent group is higher than that of the immunocompromised group. These imaging features might be associated with the strong local inflammation of the lesion in the immunocompetent group, which leads to more necrotic tissue and more exudates filling the alveolar cavity. The non-statistically significant difference in the incidence of pneumonia-like consolidation between the two groups, which may be associated with the small number of cases in this study.

*Cryptococcus* treatment regimens mainly include medical and surgical treatment. Depending on disease severity and occurrence of cryptococcal meningoencephalitis, fluconazole alone or combined with amphotericin B and flucytosine can be selected. Surgical resection could be the treatment of choice for isolated lesions that persist after medical treatment. Antifungal adjuvant therapy is recommended after surgery [18, 19]. In this study, most of the patients responded well to conservative treatment, their symptoms were relieved, the chest CT lesions were significantly reduced after reexamination, and the CSF test turned negative. Of the 7 patients (8%) who underwent surgery, 5 were admitted to the department of thoracic surgery for lung nodules and received thoracic resection of lung nodules. Four of them were given antifungal adjuvant therapy after the operation. All 5 patients were followed up without recurrence or central nervous system transmission. Another 2 cases received surgical resection after failed antifungal conservative treatment, of which 1 case reported mucinous adenocarcinoma after surgery.

In summary, prompt and accurate diagnosis of *cryptococcus* is an important prerequisite for a good prognosis. Cryptococcal infection not only occurs in patients with compromised immune function but also in people with intact immune function. Its clinical symptoms lack specificity and imaging findings are not quite typical. So the detection of CrAg in serum and CSF is of great significance in diagnosis and therapeutic observation.

## Supplementary Information

**Additional file 1**. The clinical data of 50 patients diagnosed with cryptococcosis in the Zhongda Hospital of Southeast University from January 2014 to May 2020 in the study.

## Data Availability

The datasets used are available from the corresponding author on reasonable request.
